# Using mechanical testing to assess texturing of prosthetic sockets to improve suspension in the transverse plane and reduce rotation

**DOI:** 10.1371/journal.pone.0233148

**Published:** 2020-06-11

**Authors:** Julia Quinlan, Vasanth Subramanian, Jessica Yohay, Brad Poziembo, Stefania Fatone

**Affiliations:** 1 Northwestern University Prosthetics-Orthotics Center, Department of Physical Medicine and Rehabilitation, Northwestern University, Chicago, Illinois, United States of America; 2 Prosthetic Design Inc., Dayton, Ohio, United States of America; University of Illinois at Urbana-Champaign, UNITED STATES

## Abstract

Creating a secure and comfortable linkage between the residual limb and prosthetic socket in persons with lower limb amputation is a critical factor for successful rehabilitation, including ambulation and other activities of daily living. Unwanted rotation within the socket can be a clinical problem for prosthesis users. One way of addressing issues experienced with transverse plane control of the socket may be through increased friction interface forces. It has been proposed that friction at the residual limb/socket interface may be increased by adding texture to interface components. Three-dimensional (3D) printing may be used to fabricate sockets with texture patterns added to the inner socket surface. Hence, the aim of this study was to investigate the effects of socket texturing on transverse plane rotation of the socket on a mock residual limb under two suspension conditions: passive suction and active vacuum. To conduct this study, we developed a mechanical testing protocol as no standardized tests currently exist to assess prosthetic sockets. Sockets with 14 different texture patterns were fabricated using the Squirt-Shape^™^ 3D printer. Textured sockets were compared to an Original Squirt-Shape (OSS) socket and a smooth thermoformed socket. Sockets were fitted with a mock residual limb and bi-axially loaded to 350 N compression with simultaneous rotation (2.5°, 5° and 7.5°) using a custom rotation assembly attached to a uniaxial hydraulic material testing system. There was a statistically significant three-way interaction between suspension, angle and texture (p < 0.0005). Torques between textured and reference sockets, for all rotation angles and both suspension conditions, were significantly different (p < 0.0005). Using newly developed testing protocols, it was demonstrated that some texture patterns significantly increased torque (i.e., resistance against unwanted rotation) in the transverse plane compared to both OSS and smooth sockets, especially for passive suction. Rotation testing of sockets may provide insight into socket design to improve suspension in the transverse plane.

## Introduction

Creating a secure and comfortable linkage between the residual limb and prosthetic socket in persons with lower limb amputation is a critical factor for successful rehabilitation, including ambulation and other activities of daily living. A secure linkage between the residual limb and socket requires some friction forces acting at the interface between them [1] to diminish relative motion. An ill-fitting socket compromises these forces, resulting in relative movement between the residual limb and socket that may cause discomfort, irritation or damage to the skin of the residual limb [2, 3]. If shear and friction become excessively large, they may cause skin damage or unwanted wear and tear of interface components, including elastomeric liners [4, 5].

The generation of sufficient interface forces to control relative motion without causing other problems continues to be a clinical challenge both in the longitudinal/axial direction as well as the transverse plane. Reducing pistoning [6–9] and improving suspension in the longitudinal plane using a variety of suspension mechanisms including elevated vacuum, passive suction, and pin-locking liners [10–14] has been investigated, with elevated vacuum suspension generally reducing pistoning more so than other forms of suspension. However, fewer attempts to address transverse plane rotation of the residual limb inside the socket have been described [6–9]. Nevertheless, it remains a clinical problem with prosthesis users reporting unwanted transverse plane rotation within the socket [10–12].

Rotational control of the prosthetic socket on the residual limb is needed during both stance and swing phases of walking. In swing phase, transverse plane gait deviations known as whips can occur due to poor suspension [13]. In stance phase, the anatomical ankle and foot act to absorb the forces that occur when the body moves over the foot as it maintains contact with the ground. Prosthetic feet do not provide the same functionality as anatomical feet, resulting in shear stress at the residual limb when walking and especially when turning [7, 14, 15]. Torsion adapters have been developed to try to minimize the shear stress experienced at the residual limb in persons with transtibial amputation [16, 17].

An alternative way of addressing issues experienced with transverse plane control of the socket may be through increased friction interface forces. It has been proposed that friction at the residual limb/socket interface can be increased by adding texture to interface components [18–21]. Three-dimensional (3D) printing, which allows for control of texture characteristics such as pattern shape, size and distribution [20], may be used to fabricate sockets with texture patterns added to the inner socket surface. One of the first applications of 3D printing using fused deposition modeling to fabricate prosthetic sockets was described by Rolock [22, 23] and later commercialized as the Squirt-Shape^™^ 3D printer (Prosthetic Design Inc., Dayton, OH). Squirt-Shape sockets with slight horizontal striations/texturing have been in clinical use for over a decade.

Previous studies [19, 21] reported an increase in static coefficient of friction of textured socket samples compared to smooth socket samples when tested according to the American Society for Testing and Materials (ASTM) standard [24]. However, kinetic coefficient of friction of textured socket samples did not exhibit the same response [21]. This finding was attributed to the standardized test set-up [24] as it does not recreate the type of engagement between material surfaces that occurs when the residual limb is compressed within the liner and socket. Hence, to investigate the effect of texturing on socket suspension forces, there is need for test set-ups that more closely mimic the behavior of the residual limb inside the socket.

Mechanical testing has the potential to offer insights regarding interface designs aimed at reducing relative motion between the socket and residual limb, yet no guidelines exist for how this may be accomplished. Previous studies modified the International Organization for Standardization (ISO) 10328 standard [25] testing methods to include assessment of prosthetic sockets [26–29], yet these tests focus on socket strength and are not helpful in the assessment of suspension forces. While it may be relatively straightforward to assess longitudinal displacement of a socket on a mock residual limb using testing conditions that resemble those experienced during ambulation or activities of daily living [20, 30, 31], assessing rotational displacement is more challenging given the cost of multi-axial material testing systems [32].

Given the above, the aim of this project was to investigate the effects of socket texturing on transverse plane rotation of the socket on a mock residual limb under two suspension conditions: passive suction and active vacuum. In order to conduct this study, we adapted a rack and pinion design by Edwards and Troy [32] and developed a mechanical testing protocol. We hypothesized (1) that sockets with novel textures would require greater torque when compared to a smooth socket and an Original Squirt-Shape (OSS) socket to achieve the same rotational displacement; and (2) that sockets with active vacuum would require a greater torque for a given rotation angle compared to sockets with passive suction. Additionally, we considered whether more aggressive texture patterns and/or those with primarily vertical pattern orientation, as compared to more subtle texture patterns and/or those with primarily horizontal pattern orientation, resulted in greater torque for a given rotation angle.

## Materials and methods

### Socket fabrication

**Fig 1:**
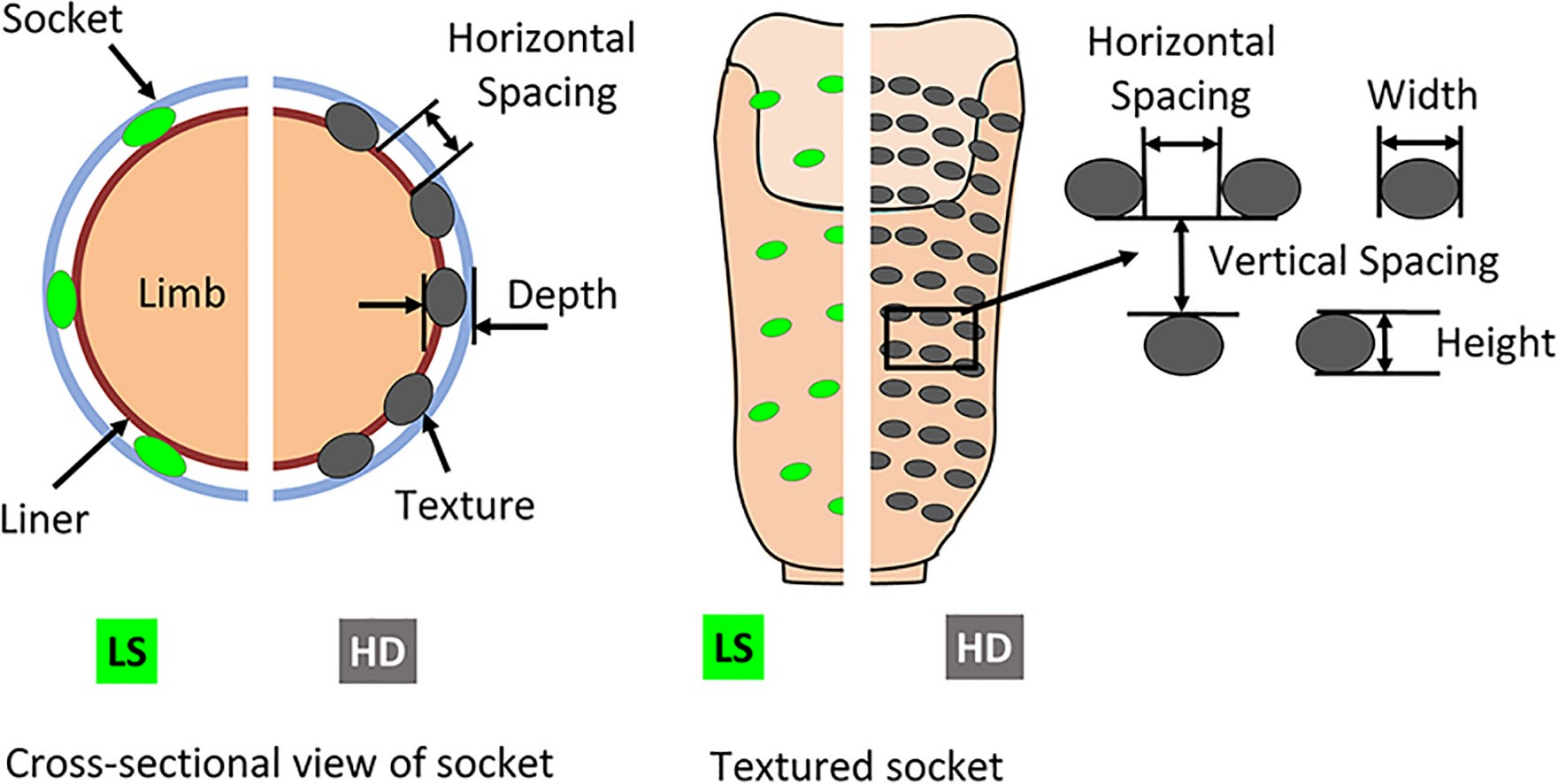
A. Texture presented in cross-section and B. interior views of 3D printed light and sparse (LS) and heavy and dense (HD) prosthetic sockets.

**Fig 2:**
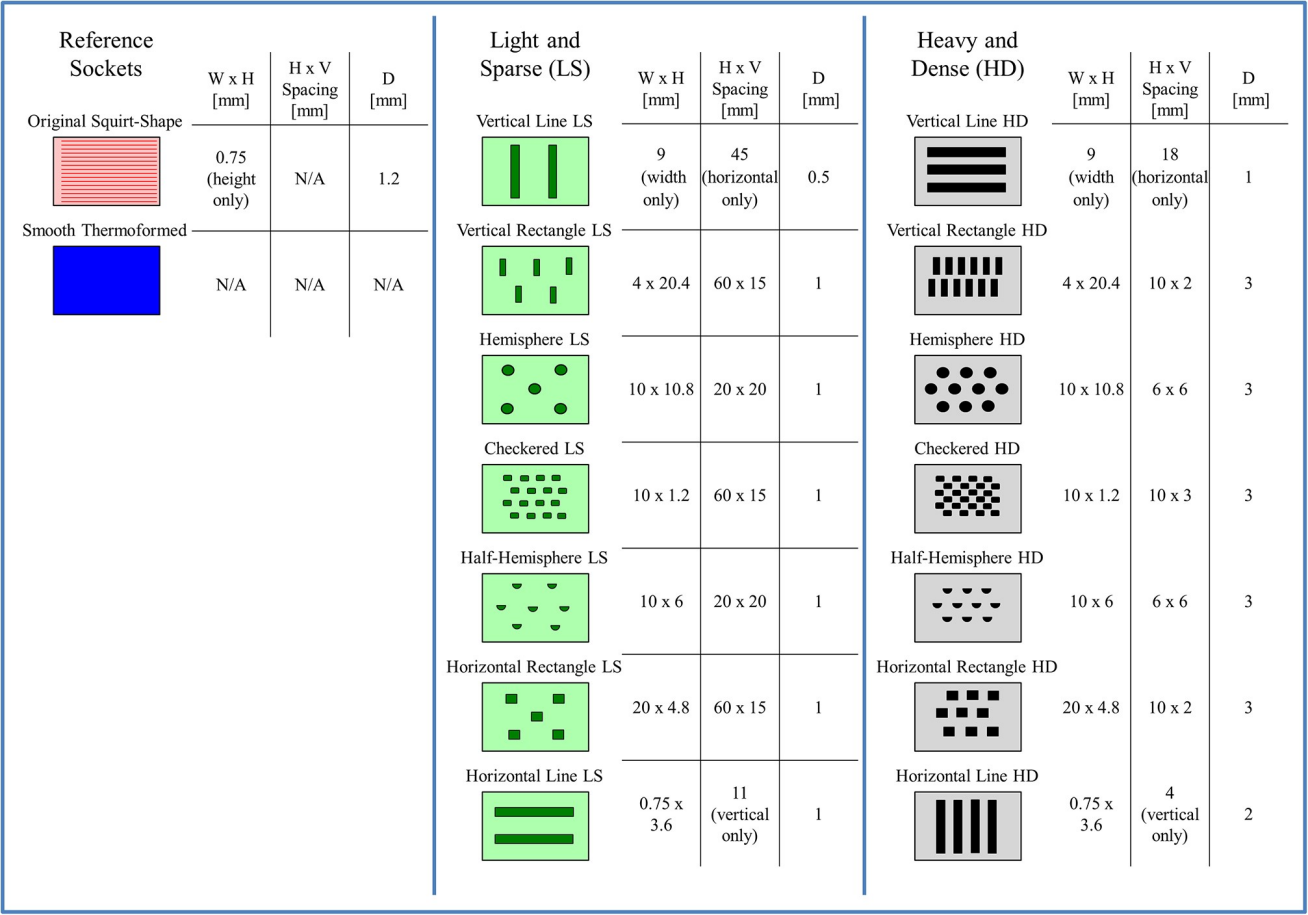
Schematic of novel texture and reference patterns assessed in this study and their respective dimensions. Textures are organized, top left to bottom right, from those with a primarily vertical orientation of texture pattern to those with a primarily horizontal orientation of texture pattern. (W x H–width and height in [mm], H x V spacing–horizontal and vertical spacing in [mm], D–depth in [mm]).

Total Surface Bearing prosthetic sockets were fabricated using polypropylene copolymer pellets (PPM-50, Prosthetic Design Inc., Dayton, OH) and a Squirt-Shape^™^ 3D Printer (Prosthetic Design Inc., Dayton, OH). A selection of seven novel texture patterns were programmed and 3D printed into the socket wall. Texture patterns included simple designs such as horizontal and vertical lines, horizontal and vertical rectangles, as well as more complicated designs such as checkered, hemisphere and half-hemisphere [21]. Two sockets were fabricated for each texture pattern (Figs 1 and 2): “light and sparse” (LS, representing a subtle version of the texture pattern with a smaller depth, d1, which is sparsely distributed) and a second version that was “heavy and dense” (HD, representing a more pronounced version of the texture pattern with a larger depth, d2, which is more densely distributed) [21].

During socket fabrication, our goal was to obtain a similar fit between the sockets with different textures and a mock residual limb. This was achieved by ensuring that there was resistance when sliding the mock residual limb into the socket while obtaining distal end contact. The volume of each socket was globally altered in the computer-aided design (CAD) software to obtain these characteristics. Estimates of socket volume from the digital CAD files indicate that heavy dense sockets had a greater socket volume (range: 1.16%–4.85%) when compared to the light sparse sockets.

Additionally, two reference sockets were fabricated to be used for comparison with novel textured sockets. An OSS socket was fabricated using the same 3D printer and the same polypropylene copolymer material as the novel textured sockets. The OSS socket had horizontal striations approximately 1.2 mm in depth and 0.75 mm in thickness [22, 23]. A smooth polypropylene socket was fabricated using standard clinical thermoforming techniques (Copoly Natural White, NorthSea Plastic, 13.5” x 13.5” (342.90mm x 342.90 mm) sheet, Part number: CPPSQ343N12, Endolite, Miamisburg, OH). Sockets were made to fit the shape of a transtibial mock residual limb, which we fabricated from dual durometer urethane (outer shell hardness 20A and inner core hardness 60A) and covered with a prosthetic liner (Evolution Standard Liner, EOC-4, Össur Americas, Foothill Ranch, CA).

### Development of testing apparatus

**Fig 3:**
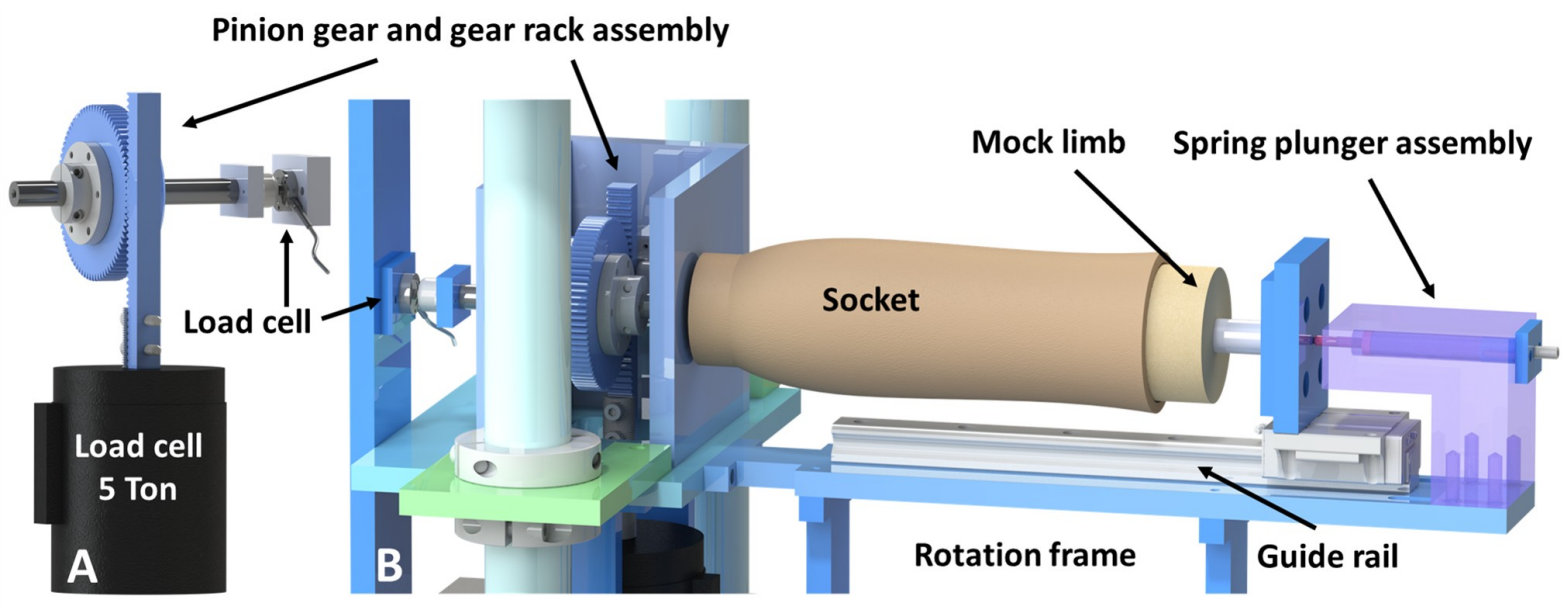
A. Pinion gear and gear rack assembly close up. B. Rotation assembly close up, transparent detail of spring plunger assembly.

Rotational testing was conducted using a uniaxial hydraulic material testing system (Instron, Norwood, MA). Assembly and fixtures needed to conduct testing were designed using CAD modeling software (SOLIDWORKS Premium 2017 x64 Edition SP 2.0, Dassault Systèmes SOLIDWORKS Corp., Waltham, MA). The transformation of linear actuation of a uniaxial material testing system into rotational movement has been described previously for torsional testing of human cadaveric proximal tibiae using a rack and pinion design [32]. Similarly, we constructed a custom rotational testing frame to translate linear loading to rotational loading of the socket around the mock residual limb, while allowing compression and rotation to be simultaneously applied to the socket and mock residual limb. Fitting parts of the assembly into each other with high precision helped avoid offset forces or moments that could potentially damage the uniaxial testing system and its load cell. The components of the testing apparatus consisted of (Fig 3) a uniaxial hydraulic material testing system (Instron, Norwood, MA), load cell (5 metric ton, model: 661.21A-02, MTS System Corporation, Minneapolis, MN) and a custom rotation assembly. The load cell was calibrated annually by the manufacturer and the resulting calibration file was used for calculations. The load cell was also tested by the manufacturer in our testing environment across multiple days to ensure reliability of the sensor.

The custom rotation assembly consisted of a custom external loading frame constructed of 6061 aluminum stock (McMaster-Carr, Elmhurst, IL), high-load steel gear rack (20 degree pressure angle, 2 feet (0.61 m) length, 16 pitch, face width 3/4" (19.05 mm), height 3/4" (19.05 mm), McMaster-Carr, Elmhurst, IL) and high-load steel pinion gear (20 degree pressure angle, 16 pitch, 80 teeth, pitch diameter 5" (127 mm), face width 3/4" (19.05 mm), McMaster-Carr, Elmhurst, IL), steel tapered-roller bearings (3/4" (19.05 mm) shaft diameter, trade No. 09067, 1-15/16" (49.21 mm) OD, McMaster-Carr Elmhurst, IL), one-piece steel thrust ball bearing (3/4" (19.05 mm) OD, open, McMaster-Carr, Elmhurst, IL), flange-mount ball transfer (heavy duty 1/2" (12.70 mm) steel ball, steel housing, 100 lb (45.36 kg) capacity, McMaster-Carr, Elmhurst, IL), 34mm wide guide rail for ball bearing carriage (440 mm in length, McMaster-Carr, Elmhurst, IL), spring plunger (6061 aluminum stock, McMaster-Carr, Elmhurst, IL) with compression spring (LC 150HK 11S, OD 0.688" (17.48 mm), hole diameter 0.720" (18.29 mm), wire diameter 0.148" (3.76 mm), Lee Spring, Brooklyn, NY), hardened 4140 alloy steel rod (3/4" (19.05 mm) diameter x 2 feet (0.61 m), McMaster-Carr, Elmhurst, IL), and additional custom and stock fixtures, shaft collars, bolts, screws and washers (McMaster-Carr, Elmhurst, IL) necessary to position the socket and mock residual limb onto the external loading frame.

The pinion gear and gear rack assembly rotation was mounted inside the aluminum housing using steel tapered-roller bearings and a steel rod and it was connected via a threaded rod matching the hole of the main load cell (Fig 3A). The piston of the uniaxial system moved up and down (linear actuation) and connected through the gear rack to rotate the pinion gear in either a clockwise or counterclockwise motion, hence rotating the socket around the mock residual limb, while the mock residual limb was held stationary. Torque *T* (Nm) was calculated using the force *F* (N) measured by the load cell and the moment arm *r*, which was the pitch radius (0.0635 m) of the pinion gear:

T=(F−f)r;

Friction *f* calculated based on pilot testing (1.60 N—1.99 N) was considered negligible compared to the measured loads, hence the torque calculations were simplified to:

T=0.0635F.

An additional compression load cell (LLB400, 2000 lb (8896.44 N) with load button cell design, FUTEK Advanced Sensor Technology, Inc., Irvine, CA) was attached at the distal end of the socket–mock residual limb rotation assembly (Fig 3A) to measure compressive forces applied on the mock residual limb and socket during rotation testing. This load cell was also calibrated annually by the manufacturer and the resulting calibration file was used for calculations. This load cell was also tested by the manufacturer in our testing environment across multiple days to ensure reliability of the sensor. The load cell was pushed via flange-mount ball transfer (heavy duty 1/2" (12.7 mm) steel ball, steel housing, 100 lb (45.36 kg) capacity, McMaster-Carr, Elmhurst, IL) such that no shearing forces contaminated compression data during rotation and it was externally powered and amplified via an external USB output kit (Model: USB520, FUTEK Advanced Sensor Technology, Inc., Irvine, CA) with external software (SENSIT Test and Measurement, version 2.5.1.0 for IDA100, IHH500, IPM650, USB, FUTEK Advanced Sensor Technology, Inc., Irvine, CA) used to collect the data. Two wireless motion trackers (MTw sensors, Xsens North America Inc., El Segundo, CA) were used to measure rotation angles. Glue and Velcro were used to attach one sensor to the distal end of the socket, and another along the anterior aspect of the mock limb on the top of the sealing sleeve. Data were tracked and collected via the MTw development kit (Awinda station, power supply, Xsens North America Inc., El Segundo, CA).

A 3 mm liner and one nylon stocking were donned over the mock residual limb before it was seated inside the socket, which had an elevated vacuum attachment plate with manifold with air fitting (EV-AP4-BK-CAUC and EV-MAN, Prosthetic Design Inc., Dayton, OH) attached to the distal end. A sealing sleeve (Harmony Sleeve, 454A7 = K, Ottobock North America, Austin, TX) was donned over the exterior of the socket and sealed proximally to the liner above the socket trim lines. Distally, electrical tape was used to seal the sleeve to the socket given that more aggressive texturing caused some leaking of air from between the liner and socket. It is not uncommon in clinical practice for tape to be used to ensure that the distal end of the sleeve seals well to the exterior of the socket. Two suspension conditions were tested: 1) passive suction, which was created by attaching a one-way valve to the manifold, and 2) active vacuum, which was created using an electronic vacuum pump (LimbLogic^™^, WillowWood, Mt. Sterling, OH) connected to the manifold. During testing the pump was set to 20 inHg (67.73 kPa).

### Testing procedure

The socket and mock residual limb were positioned horizontally into the external testing frame and pre-compression of 350 N was applied to ensure the mock residual limb was seated inside the socket. The socket and mock residual limb were then secured by the spring plunger and a set of screws, maintaining the 350 N pre-compression during rotational movement of the socket around the mock residual limb. Compression of 350 N (approximately 36 kg) resembles partial body weight on one limb of a 72 kg person and was determined during pilot testing to be the maximum that could be applied. Higher compression loads resulted in large, potentially damaging offset moments to the Instron. Tuning of the Instron for displacement control was performed according to manufacturer instructions [33]. Tuning of the Instron for load control was not performed due to the complexity of the testing frame and to minimize the risk of potential damage to the testing system.

To assess frictionless movement of our rotational testing set-up, we conducted pilot rotation tests using the same testing protocol with the socket mounted but without the mock residual limb attached. Ten trials per rotation angle were performed. To assess repeatability, tests were repeated using the same method, user, environmental conditions, and calibrated load cells. Average friction of the set-up was recorded as 1.60 (±0.72) N, 1.79 (±0.81) N and 1.99 (±0.82) N for rotation angles 2.5°, 5° and 7.5°, respectively. Hence, the set-up was deemed nearly frictionless and with little variability.

**Fig 4:**
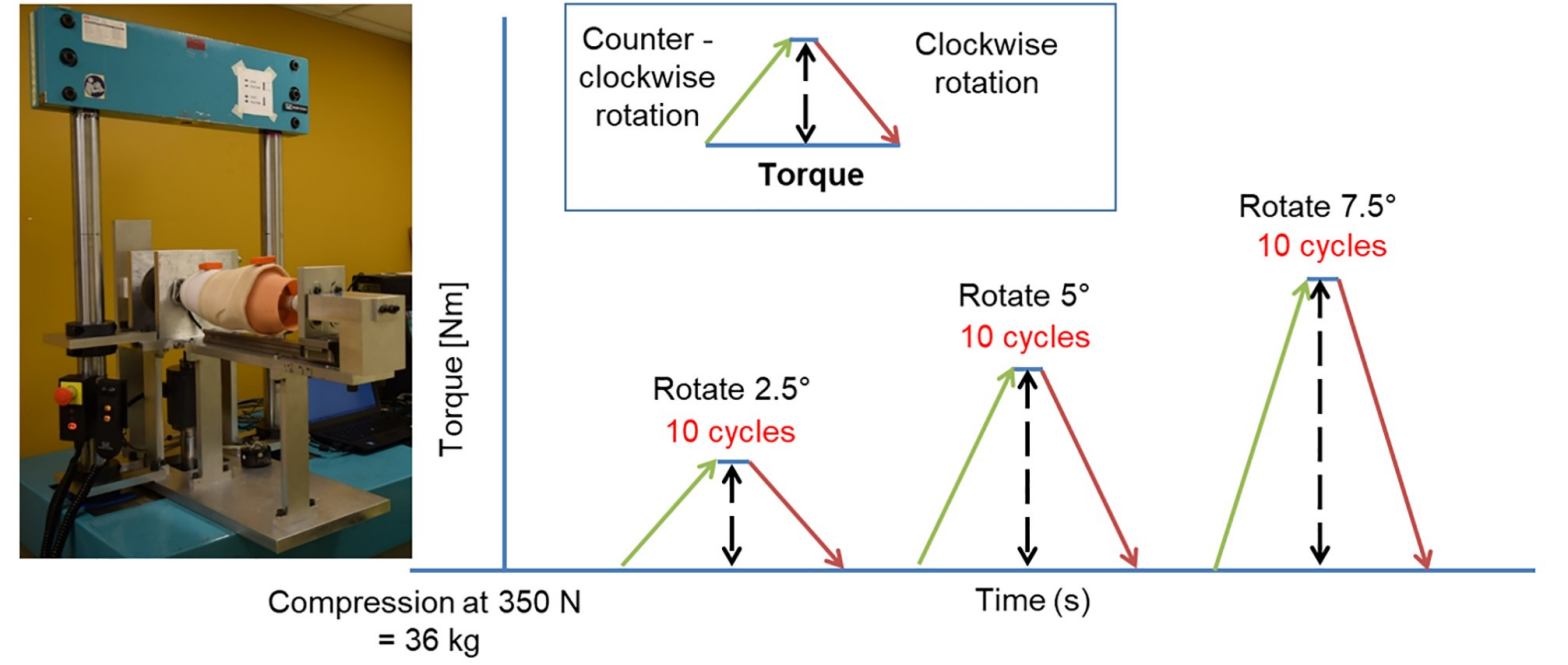
Protocol used for rotation testing: After pre-compressing the socket and mock residual limb to 350 N, the socket was rotated around the mock residual limb and subjected to six trials of ten cycles at three increasing rotation angles.

Each socket was subjected to six trials consisting of ten cycles at each of three rotation angles in the following order: 2.5°, 5° and 7.5°. These values were informed by previous studies that reported 8° of external rotation of the shank over the foot and 3° to 8.5° relative internal-external rotation between the pelvis and socket during the stance phase of walking of persons with a transfemoral prosthesis [34] and rotation of 0.8° to 10.8° of the gel liner over the limb relative to the transtibial socket [35]. Pilot testing determined that a rotation angle of 10° was damaging to the liner and produced extremely high torques, hence we selected 7.5° as the maximum rotation angle. During testing at the first rotation angle of 2.5° as an example, the socket at the distal end was cyclically rotated ten times, first clockwise to 2.5° and then counterclockwise to 0°. Fig 4 depicts the rotational protocol used for testing.

Despite pre-compression of the socket and mock residual limb, additional settling of the mock residual limb inside the socket occurred during the first trial, hence only five trials of data from each socket were included in the final analysis. We avoided testing the same liner and the same mock residual limb more than once per day in order to avoid any unwanted motion that may occur due to flow characteristics of the elastomeric materials used in the mock residual limb and liner [36, 37].

### Data analysis

Torques between the socket and mock residual limb and the corresponding rotation angles were recorded and analyzed using custom MATLAB (MathWorks, Natick, MA) code. Statistical analyses were performed using SPSS version 25 (IBM, Armonk, NY) with torque as the dependent variable having three rotation angles (2.5°, 5° and 7.5°). We assessed the torque of 16 different sockets: 2 reference sockets (smooth thermoformed and OSS sockets) and 14 novel textured sockets. Each socket was tested six times in rotation under two suspension conditions: passive suction suspension using a one-way valve (OV) and active vacuum suspension (VAC). Three independent variables were analyzed: texture (a between socket measure), and suspension (a within socket, repeated measure) and rotation angle (a within socket, repeated measure).

A three-way mixed analysis of variance “between-within-within” (ANOVA BWW) was conducted first. Torque data were not normally distributed as indicated by the Shapiro–Wilk test. Hence, we normalized torque data using a two-step rank procedure [38]. We proceeded with ANOVA analyses since they are generally robust to violations of homogeneity if group sizes are equal, which they were in this case. ANOVA BWW was used to assess three-way and two-way interactions, main effects and simple comparisons, conducted between suspension conditions for individual texture types separately for each rotation angle. Statistical significance was set at a Bonferroni-adjusted alpha level of 0.0083.

Significant interactions and main effects among all the independent variables were found, hence we performed additional Welch one-way ANOVAs to assess our hypotheses. Games-Howell post-hoc analysis for pairwise comparisons between novel and reference textures was conducted, analyzing six torques (three at each rotation angle for each suspension condition). Critical alpha was initially set at 0.05, then corrected to a Bonferroni-adjusted alpha level of 0.003125.

## Results

### Torque results

**Fig 5:**
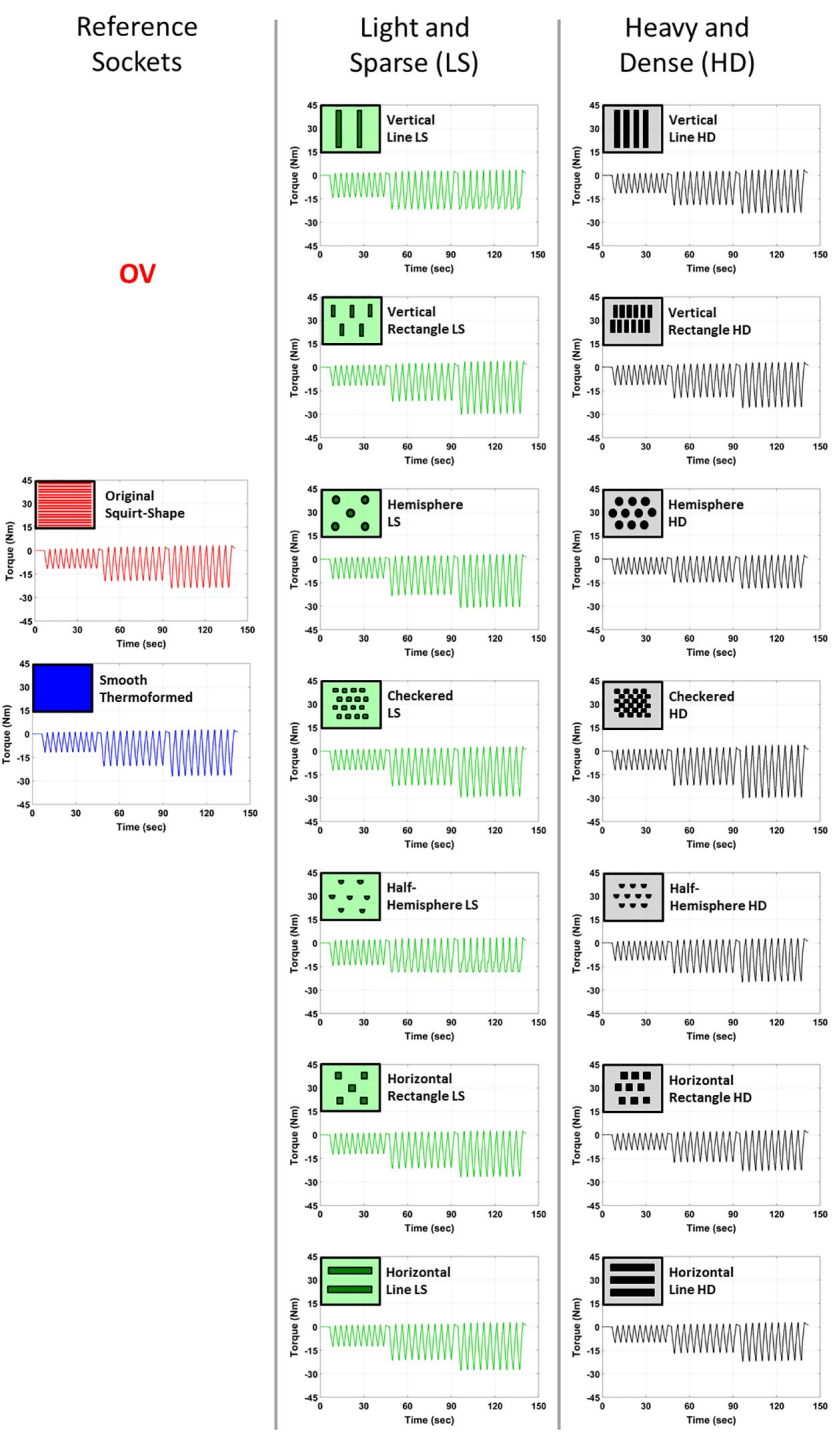
Mean torque for passive suction suspension with one-way valve (OV).

**Fig 6:**
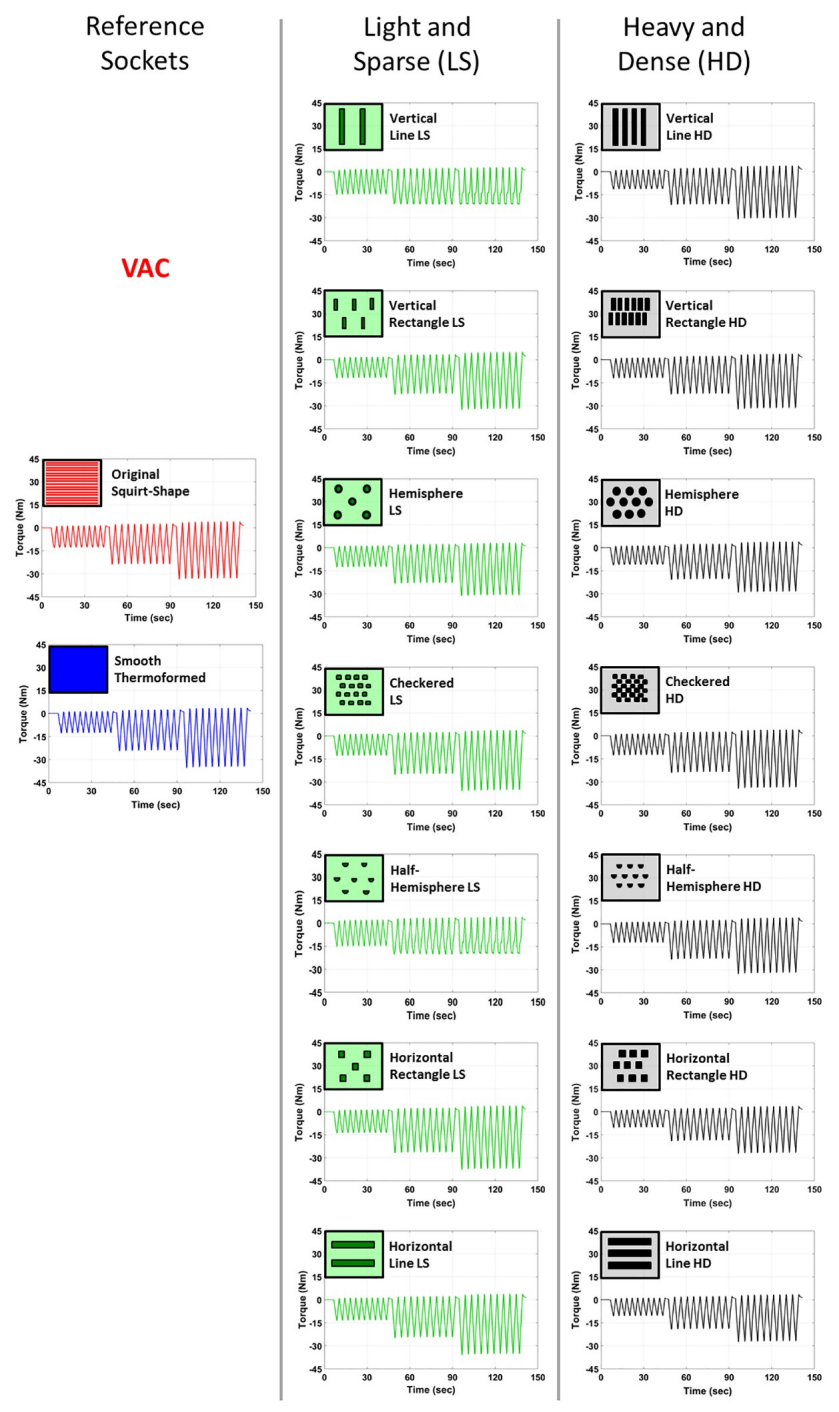
Mean torque for active vacuum with pump at 20 inHg (67.73 kPa) (VAC).

Figs 5 and 6 illustrate the mean torque in the transverse plane over 9 cycles for all 16 sockets at the three rotation angles and under two suspension conditions. The range of torques for all sockets increased with increasing rotation angle and was greater for active vacuum suspension than passive suction at every rotation angle:

- at 2.5° rotation angle: OV: 10.387 to 16.060 Nm and VAC: 10.894 to 16.645 Nm;
- at 5° rotation angle: OV: 17.693 to 26.055 Nm and VAC: 20.960 to 28.545 Nm; and
- at 7.5° rotation angle: OV: 20.473 to 37.266 Nm and VAC: 24.801 to 43.030 Nm;

### Interactions and main effects

There was a statistically significant three-way interaction between suspension, rotation angle and texture, F(24.117, 1131.908) = 353.280, p < 0.0005, partial η2 = .883, ε = .804. There was also a statistically significant (p < 0.0005) simple two-way interaction between suspension and rotation angle for all texture types, and a statistically significant (p < 0.0005) simple-simple main effect of suspension on all measured torques for all rotation angles. More detailed statistical results are summarized in the S1 Appendix.

### Comparing novel textures to reference sockets

Torques in the transverse plane between texture and reference sockets for all rotation angles and both suspension conditions were significantly different at p < 0.0005. Specifically:

- for 2.5° rotation angle: OV: Welch’s F(15, 264.595) = 1366.253; VAC: Welch’s F(15, 265.288) = 3232.694;
- for 5° rotation angle: OV: Welch’s F(15, 265.254) = 1956.274; VAC: Welch’s F(15, 264.986) = 1977.798; and
- for 7.5° rotation angle: OV: Welch’s F(15, 264.639) = 1727.067; VAC: Welch’s F(15, 264.967) = 2246.391.

Table 1 summarizes the results of the Games-Howell post-hoc analysis. Novel texturing significantly increased torques compared to the reference sockets at p < 0.0005.

**Table 1:** Absolute Mean and Standard Deviation (SD) for torque at each rotation angle for both suspension conditions (OV: Passive suction with one-way valve; VAC: Active vacuum suspension at 20 inHg (67.73 kPa); Nm: Newton meter; LS: Light and sparse; HD: Heavy and dense).

Socket	2.5°		5°		7.5°	
	OV [Nm]	VAC [Nm]	OV [Nm]	VAC [Nm]	OV [Nm]	VAC [Nm]
Smooth Thermoformed	12.786 (0.118) ‡	14.190 (0.178)	21.771 (0.182) ‡	26.591 (0.192) ‡	29.138 (0.375) ‡	38.563 (0.364) ‡
Original Squirt-Shape (OSS)	12.418 (0.164) ¥	14.518 (0.112)	21.104 (0.165)	25.732 (0.299) ¥	27.337 (0.283) ¥	35.866 (0.564) ¥
Vertical Line LS	15.129 (0.164) † ‡	15.685 (0.170) † ‡	25.017 (1.555) † ‡	23.613 (0.235) ¥ #	25.655 (0.910) ¥ #	26.819 (0.984) ¥ #
Vertical Rectangle LS	14.137 (0.728) † ‡	13.483 (0.153) ¥ #	25.906 (1.768) † ‡	25.385 (0.170) ¥ #	31.419 (2.056) † ‡	36.272 (0.345) ¥
Hemisphere LS	14.446 (0.137) † ‡	13.687 (0.098) ¥ #	26.055 (0.603) † ‡	25.002 (0.113) ¥ #	33.371 (0.839) † ‡	33.084 (0.415) ¥ #
Checkered LS	13.567 (0.102) † ‡	10.894 (0.904) ¥ #	23.570 (0.202) † ‡	22.491 (0.232) ¥ #	30.827 (0.214) † ‡	30.829 (0.616) ¥ #
Half-hemisphere LS	16.060 (0.526) † ‡	16.645 (0.542) † ‡	21.646 (0.307) ‡	23.373 (0.744) ¥ #	23.303 (0.476) ¥ #	24.801 (2.586) ¥ #
Horizontal Rectangle LS	13.874 (0.091) † ‡	15.206 (0.117) † ‡	22.740 (0.137) † ‡	28.545 (0.730) † ‡	29.062 (0.403) ‡	43.030 (1.683) † ‡
Horizontal Line LS	13.732 (0.885) † ‡	14.846 (0.095) † ‡	22.351 (0.154) † ‡	27.249 (0.233) † ‡	30.115 (0.203) † ‡	40.051 (0.527) † ‡
Vertical Line HD	12.442 (0.166) ¥	12.758 (0.084) ¥ #	23.165 (0.158) † ‡	23.813 (0.162) ¥ #	34.335 (0.706) † ‡	32.878 (0.446) ¥ #
Vertical Rectangle HD	13.082 (0.083) † ‡	13.031 (0.076) ¥ #	24.690 (0.268) † ‡	24.316 (0.103) ¥ #	37.266 (1.503) † ‡	34.109 (0.219) ¥ #
Hemisphere HD	11.430 (0.288) ¥ #	12.454 (0.096) ¥ #	17.693 (1.048) ¥ #	23.058 (0.129) ¥ #	20.473 (1.856) ¥ #	31.807 (0.244) ¥ #
Checkered HD	13.541 (0.262) † ‡	14.102 (0.168) #	24.009 (0.322) † ‡	26.065 (0.187) ‡ ¥	31.746 (0.423) † ‡	37.444 (0.295) ‡ ¥
Half-hemisphere HD	12.195 (0.405) ¥	13.332 (0.076) ¥ #	20.625 (0.163) ¥ #	24.678 (0.118) ¥ #	28.039 (0.245) ‡ ¥	34.887 (0.252) ¥ #
Horizontal Rectangle HD	11.188 (0.266) ¥ #	11.332 (0.390) ¥ #	20.048 (0.185) ¥ #	20.960 (1.109) ¥ #	26.141 (0.503) ¥ #	29.670 (0.546) ¥ #
Horizontal Line HD	10.387 (0.913) ¥ #	12.061 (0.135) ¥ #	19.291 (0.269) ¥ #	21.181 (0.762) ¥ #	24.987 (0.463) ¥ #	29.387 (0.795) ¥ #

^†^ Significantly greater torque than smooth socket, p < 0.0005.

^‡^ Significantly greater torque than OSS socket, p < 0.0005.

^¥^ Significantly smaller torque than smooth socket, p < 0.0005.

^#^ Significantly smaller torque than OSS socket, p < 0.0005.

### Comparing passive suction and active vacuum (Table 1)

For passive suction across all rotation angles, greater torques were observed for 10 of 14 novel textured sockets when compared to the smooth socket, and 11 of 14 when compared to the OSS socket. When active vacuum was applied, fewer novel textured sockets produced increased torques compared to the two reference sockets (4 of 14 compared to the smooth socket, and 5 of 14 compared to the OSS socket).

For the smallest rotation angle of 2.5° with passive suction, 9 of 14 novel textured sockets had significantly greater torques than the smooth socket. The same 9 novel textured sockets and the smooth socket had significantly greater torques than the OSS socket. Additionally, 5 of 14 novel textured sockets and the OSS socket had significantly smaller torques than the smooth socket and 3 of 14 novel textured sockets had significantly smaller torques than the OSS socket. With active vacuum, 4 of 14 novel textured sockets had significantly greater torques than both the smooth and OSS sockets. Additionally, 9 of 14 novel textured sockets had significantly smaller torques than the smooth socket and 10 of 14 novel textured sockets had significantly smaller torques than the OSS socket.

For the next rotation angle of 5° with passive suction, 9 of 14 novel textured sockets had significantly greater torques than the smooth socket. Ten of 14 novel textured sockets and the smooth socket had significantly greater torques than the OSS socket. Additionally, 4 of 14 novel textured sockets had significantly smaller torques than both the smooth and OSS sockets. With active vacuum, 2 of 14 novel textured sockets had significantly greater torques than the smooth socket. Three of 14 novel textured sockets and the smooth socket had significantly greater torques than the OSS socket. Additionally, 12 of 14 novel textured sockets and the OSS socket had significantly smaller torques than the smooth socket and 11 of 14 novel textured sockets had significantly smaller torques than the OSS socket.

For the largest rotation angle of 7.5° N with passive suction, 7 of 14 novel textured sockets had significantly greater torques than the smooth socket. Nine of 14 novel textured sockets and the smooth socket had significantly greater torques than the OSS socket. Additionally, 6 of 14 novel textured sockets and the OSS socket had significantly smaller torques than the smooth socket and 5 of 14 novel textured sockets had significantly smaller torques than the OSS socket. With active vacuum, 2 of 14 novel textured sockets had significantly greater torques than the smooth socket and 3 of 14 novel textured sockets and the smooth socket had significantly greater torques than the OSS socket. Additionally, 12 of 14 novel textured sockets and the OSS socket had significantly smaller torques than the smooth socket and 10 of 14 novel textured sockets had significantly smaller torques than the OSS socket.

### Comparing type of novel texturing

**Fig 7:**
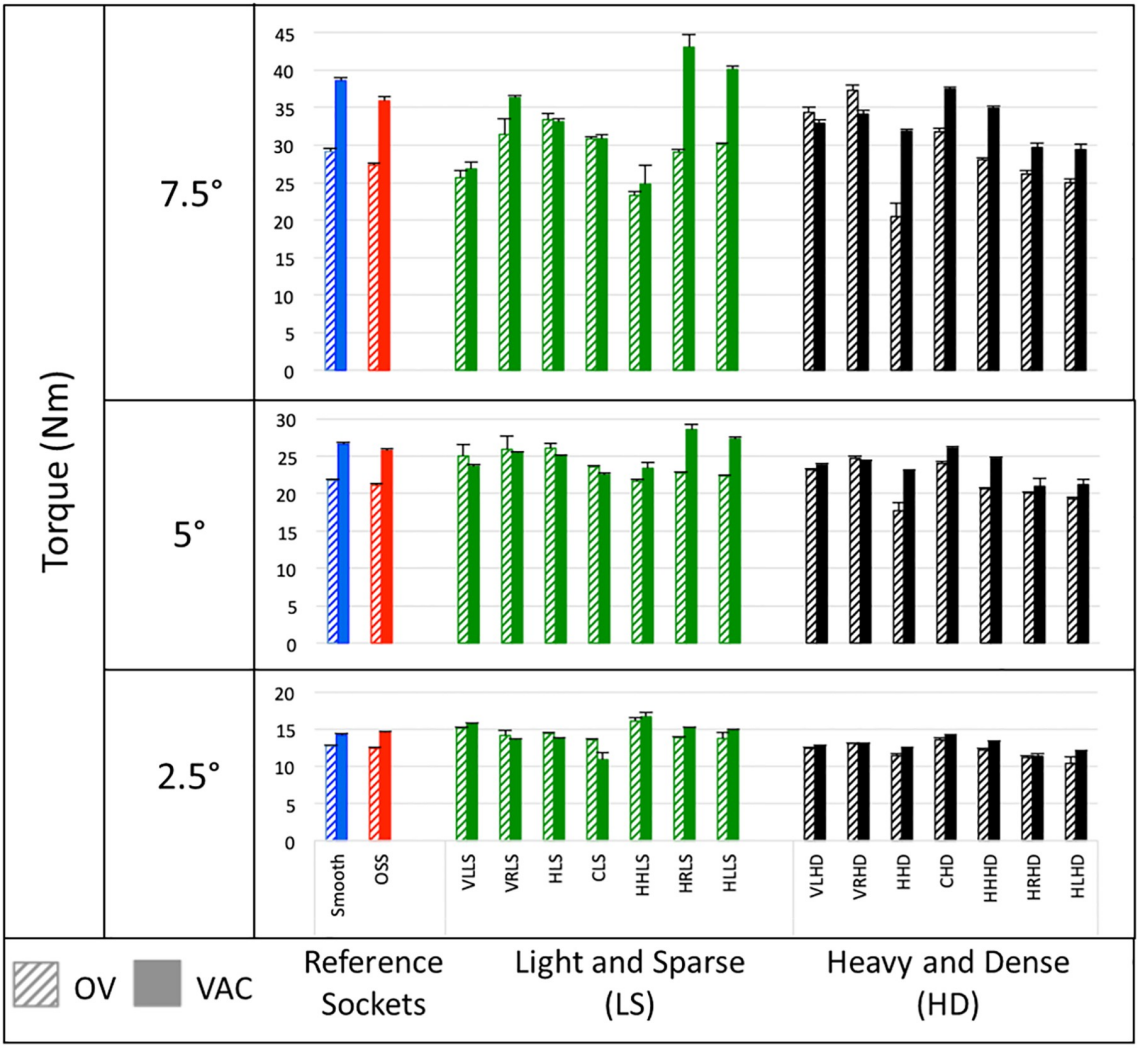
Mean torques in the transverse plane for passive suction suspension with one-way valve (OV) and active vacuum with pump at 20 inHg (67.73 kPa) (VAC). Textures are organized from left to right from those with a primarily vertical orientation of texture pattern to those with a primarily horizontal orientation of texture pattern. Also, heavy and dense (HD) patterns are grouped to the right of light and sparse (LS) patterns.

Fig 7 illustrates mean torques for the reference sockets compared to the novel textured sockets for each of the three rotation angles (2.5°, 5° and 7.5°) including the effect of suspension system on the measured torque. Greater torques in the transverse plane were observed for the smooth socket when compared to the OSS socket in all but the active vacuum condition at the lowest rotation angle (2.5°). Greater torques in the transverse plane were observed under active vacuum for almost all of the novel textured sockets when compared to passive suction. There was no difference in torque based on orientation of texture patterns from vertical to horizontal. Largest torques were observed for two light and sparse texture patterns (Horizontal Rectangle LS and Horizontal Line LS) as compared to rest of the texture patterns in the active vacuum condition.

## Discussion

The aim of this study was to investigate the effect of socket texturing on transverse plane rotation of the socket on a mock residual limb under two suspension conditions: passive suction and active vacuum. Rotational testing demonstrated that socket texturing influences socket suspension in the transverse plane. Our hypothesis that novel textures would increase torques in the transverse plane compared to smooth and OSS sockets was partially supported. While most of the textures demonstrated increased torques in the transverse plane compared to the smooth and OSS sockets in the passive suction condition, fewer novel textures demonstrated increased torques compared to the smooth and OSS sockets under active vacuum. Our hypothesis that active vacuum would provide increased torque for a given rotation angle compared to passive suction was supported. Increased torques were observed under active vacuum for almost all of the sockets compared to passive suction at all rotation angles.

Our results suggest that there was no difference in torque between aggressive texture patterns and/or those with primarily vertical pattern orientation when compared to more subtle texture patterns and/or those with primarily horizontal pattern orientation. On the contrary, two of the light and sparse sockets with texture patterns that had a primarily horizontal orientation–Horizontal Rectangle LS and Horizontal Line LS–resulted in the highest torque at 7.5° rotation angle under active vacuum. It is possible that this may be in part because the mock residual limb was insufficiently compliant to fully engage the height of the aggressive textures. Furthermore, based on the socket volume estimated from digital CAD files, socket volume was greater for the heavy dense sockets than the light sparse sockets in order to fit onto the mock residual limb, and this may similarly have contributed to less engagement with the aggressive textures. Unfortunately, our exploration of texture patterns was limited by the subjective rank order applied to texture orientation and the combination of factors compared. Future studies would benefit from a more systematic assessment and less subjective categorization of different texture dimensions and orientations.

New methods were developed to assess socket suspension in the transverse plane (i.e., resistance to rotation) given that standardized tests are not available. The rotation testing protocol we developed to assess socket suspension in the transverse plane has merit based on the collected data. Firstly, changes in torque scaled systematically with the rotation angles applied such that the lowest rotation angle resulted in the least amount of torque and the highest rotation angle resulted in the most amount of torque. Additionally, the results demonstrated little variability in torque across the 10 cycles measured within each rotation angle.

Our rotation testing protocol resembles partial body weight on one limb of a 72 kg person, hence, for ease of comparison to previously reported studies, we have normalized torque data by a mass of 72 kg. Normalized torque collected under passive suction ranged from 0.148 ±0.013 to 0.229 ±0.007 Nm/kg for 2.5° rotation angle, from 0.253 ± 0.014 to 0.372 ± 0.008 Nm/kg for 5° rotation angle, and from 0.292 ± 0.026 to 0.532 ± 0.021 Nm/kg for 7.5° rotation angle. Torque collected under 20 inHg (67.73 kPa) active vacuum ranged from 0.156 ±0.012 to 0.238 ± 0.007 Nm/kg for 2.5° rotation angle, from 0.299 ± 0.015.84 to 0.408 ± 0.010 Nm/kg for 5° rotation angle, and from 0.354 ± 0.036 to 0.615 ± 0.024 Nm/kg for 7.5° rotation angle.

Our normalized torque results for 2.5° rotation angle under passive suction are comparable to transverse plane moments during walking reported in persons with transtibial amputation [7, 14, 15, 39]. In three persons with unilateral transtibial amputation who wore different suspension mechanisms (gel liner and sleeve; gel liner and pin lock; and pelite liner, socks and cuff), Neumann et al. [15] used a load cell mounted into the prosthesis and reported that normalized transverse plane distal socket peak moments ranged from 0.046 ±0.006 Nm/kg to 0.140 ± 0.022 Nm/kg for walking with the prosthetic foot to the inside of a circular path. Segal et al. [14, 39] and Heitzmann et al. [7] compared the effect of a torsion adapter and rigid pylon on transverse plane rotation moments. Segal et al. [14, 39] studied ten unilateral transtibial prosthesis users who wore liners and sleeves (passive suction) or pin-locking liners. They reported peak knee rotation moments that ranged from 0.024 ± 0.015 Nm/kg to 0.128 ± 0.037 Nm/kg with a torsion adapter and 0.026 ± 0.027 Nm/kg to 0.157 ± 0.043 Nm/kg with a rigid pylon for walking with the prosthetic leg to the inside of a 1m radius circular path. Heitzmann et al. [7] studied ten unilateral transtibial prosthesis users who wore various suspension mechanisms (including foam liner; pin lock; and passive suction). They reported peak knee rotation moments of 0.02 ± 0.02 to 0.11 ± 0.05 Nm/kg with a torsion adaptor and 0.02 ± 0.03 to 0.10 ± 0.05 Nm/kg with a rigid pylon for the prosthetic leg during an unplanned spin turn. We were unable to find similar transverse plane moment data for subjects with transtibial amputation using active vacuum suspension.

This study focused on socket rotation around a mock transtibial residual limb. While transverse plane rotation of the socket can be an issue for persons with transtibial amputation, causing discomfort, pain and/or soft tissue damage, issues with socket rotation around the residual limb are more prevalent in persons with transfemoral amputation [40] given the more rounded cross-section of the thigh and the deep seated position of the femur. Therefore, the rotation testing developed in this study should be replicated using a transfemoral mock residual limb. Although few studies report on unwanted socket rotation, it remains a clinical problem with some prosthetic manufacturers trying to address this issue with the use of fabric gripping strips (Uni-Grip, ST&G Corporation, Brea, CA) attached to the inside of the socket to help prevent rotation as well as the use of torsion adaptors to minimize the transfer of transverse plane rotation moments to the residual limb [7, 14–16, 39, 41].

The results of our study suggest that texturing may be used to improve socket suspension in the transverse plane and reduce unwanted socket rotation compared to the smooth thermoformed and OSS sockets especially under passive suction. The greater torques produced by the novel textured sockets reflects higher resistance to unwanted rotation compared to the reference sockets, thus improving socket suspension in the transverse plane. Generally, resistance against unwanted rotation was improved to a lesser degree under active vacuum compared to passive suction. For example, 10 out of 14 textures outperformed the smooth socket under passive suction and 4 out of 14 textures outperformed the smooth socket under active vacuum. Similarly, under passive suction 11 out of 14 textures outperformed the OSS socket and 4 out of 14 textures outperformed the OSS socket under active vacuum. Our results suggest that texturing of a socket with passive suction improves suspension in the transverse plane producing torque that is comparable to a smooth socket with active vacuum. Texturing of the socket was expected to increase friction between the socket/liner and residual limb and we observed increases in the resistance against unwanted rotation with certain textures, resulting in increased torque. What remains unknown is whether such texturing is helpful or not *in vivo*. It remains to be demonstrated if the more promising textures identified in this study (e.g. Horizontal Rectangle LS, Checkered HD, Half-Hemisphere LS and Horizontal Line LS) could be worn comfortably by actual prosthesis users and whether or not they would increase liner wear and tear.

Limitations of this study include both the extent to which the mock residual limb resembled that of a human residual limb and the extent to which the testing protocol simulates the range of loading conditions a prosthesis user would experience every day. With respect to the mock residual limb, the outer shell was made from urethane of 20A shore hardness, which is higher than the shore hardness reported for skin and subcutaneous tissues [42, 43]. A higher shore hardness was used in order to ensure durability of the mock residual limb given that an initial mock residual limb with outer urethane shell of 10A shore hardness delaminated during pilot testing. Additionally, the mock residual limb does not change shape relative to the socket, so suspension is never compromised as it might be *in vivo* when muscles contract. With respect to the test set-up and protocol we developed, it was limited to bi-axial loading, where compression and rotation were applied simultaneously. Even though bi-axial testing is more complex than the uniaxial testing typically used when evaluating sockets [20, 26–31] it does not fully simulate the range of loading directions and moments a prosthesis user would experience during every day activities. Given these limitations, our results represent a best case scenario with respect to maintaining suspension, and likely overestimates the torques that might occur at 5° and 7.5° rotation angles when adding texture to 3D printed sockets worn by persons with transtibial amputation. Additionally, static failure tests are required to assess whether adding texture to sockets compromises structural integrity and human subjects testing is required to assess whether adding texture to sockets improves suspension without causing user discomfort.

The results of this study apply only to the particular texture patterns and dimensions that we 3D printed into rigid polypropylene copolymer sockets using the Squirt-Shape^™^ 3D Printer. Different texture patterns or applying texture in a different manner (e.g. to flexible inner sockets or liners) might improve suspension in the transverse plane to a greater extent than what was evident in this study and deserve further attention.

## Conclusions

Mechanical testing protocols were developed to investigate the effect of socket texturing on transverse plane rotation of the socket on a mock residual limb. Results indicated that using the Squirt-Shape^™^ 3D Printer to add texture to polypropylene copolymer sockets significantly increased torque in the transverse plane compared to both a smooth and Original Squirt-Shape socket especially for passive suction. Increased torque relates to higher resistance against unwanted rotation, thus improving socket suspension in the transverse plane.

## Supporting information

S1 Appendix Statistical analysis results. (PDF)
