# Correction: Using mechanical testing to assess texturing of prosthetic sockets to improve suspension in the transverse plane and reduce rotation

**DOI:** 10.1371/journal.pone.0269580

**Published:** 2022-06-02

**Authors:** Julia Quinlan, Vasanth Subramanian, Jessica Yohay, Brad Poziembo, Stefania Fatone

After publication of this article, the authors determined they were incorrect in the application of the Bonferroni correction and inadvertently applied it by level rather than tests conducted. In reviewing the SPSS output files, they discovered that the Bonferroni correction was already applied as part of the ANOVA BWW analysis. Hence, they did not need to apply a manual adjustment for multiple comparisons to the ANOVA BWW as is reported in the article. The changes to the critical alphas do not change reporting of the results.

In the Data analysis subsection of the Materials and methods, there is an error in the last sentence of the second paragraph. The correct sentence is: A Bonferroni correction was applied within SPSS for the ANOVA BWW analysis, and critical alpha set at 0.05.

In the Data analysis subsection of the Materials and methods, the authors do not report the critical alpha for the Welch one-way ANOVAs. The authors provide the following additional information: For the Welch one-way ANOVAs, a Bonferroni correction was applied as 0.05/6 as 6 one-way Welch ANOVAs were conducted. The critical alpha for the Welch one-way ANOVAs was set at 0.0083.

In the Data analysis subsection of the Materials and methods, the Bonferroni correction for the Games-Howell post-hoc analyses should be reported as 0.05/30 as there were 30 pairwise comparisons conducted for each Welch one-way ANOVA. There is an error in the last sentence of the third paragraph. The correct sentence is: Critical alpha was initially set at 0.05, then corrected to a Bonferroni-adjusted alpha level of 0.00167.

Three symbols denoting a significant difference are missing from the first two rows of Table 1. Please see the correct Table 1 here.

**Table 1:** Absolute Mean and Standard Deviation (SD) for torque at each rotation angle for both suspension conditions (OV: passive suction with one-way valve; VAC: active vacuum suspension at 20 inHg (67.73 kPa); Nm: Newton meter; LS: light and sparse; HD: heavy and dense).

Socket	2.5°		5°		7.5°	
	OV [Nm]	VAC [Nm]	OV [Nm]	VAC [Nm]	OV [Nm]	VAC [Nm]
Smooth Thermoformed	12.786 (0.118) ‡	14.190 (0.178) #	21.771 (0.182) ‡	26.591 (0.192) ‡	29.138 (0.375) ‡	38.563 (0.364) ‡
Original Squirt-Shape (OSS)	12.418 (0.164) ¥	14.518 (0.112) †	21.104 (0.165) ¥	25.732 (0.299) ¥	27.337 (0.283) ¥	35.866 (0.564) ¥
Vertical Line LS	15.129 (0.164) † ‡	15.685 (0.170) † ‡	25.017 (1.555) † ‡	23.613 (0.235) ¥ #	25.655 (0.910) ¥ #	26.819 (0.984) ¥ #
Vertical Rectangle LS	14.137 (0.728) † ‡	13.483 (0.153) ¥ #	25.906 (1.768) † ‡	25.385 (0.170) ¥ #	31.419 (2.056) † ‡	36.272 (0.345) ¥
Hemisphere LS	14.446 (0.137) † ‡	13.687 (0.098) ¥ #	26.055 (0.603) † ‡	25.002 (0.113) ¥ #	33.371 (0.839) † ‡	33.084 (0.415) ¥ #
Checkered LS	13.567 (0.102) † ‡	10.894 (0.904) ¥ #	23.570 (0.202) † ‡	22.491 (0.232) ¥ #	30.827 (0.214) † ‡	30.829 (0.616) ¥ #
Half-hemisphere LS	16.060 (0.526) † ‡	16.645 (0.542) † ‡	21.646 (0.307) ‡	23.373 (0.744) ¥ #	23.303 (0.476) ¥ #	24.801 (2.586) ¥ #
Horizontal Rectangle LS	13.874 (0.091) † ‡	15.206 (0.117) † ‡	22.740 (0.137) † ‡	28.545 (0.730) † ‡	29.062 (0.403) ‡	43.030 (1.683) † ‡
Horizontal Line LS	13.732 (0.885) † ‡	14.846 (0.095) † ‡	22.351 (0.154) † ‡	27.249 (0.233) † ‡	30.115 (0.203) † ‡	40.051 (0.527) † ‡
Vertical Line HD	12.442 (0.166) ¥	12.758 (0.084) ¥ #	23.165 (0.158) † ‡	23.813 (0.162) ¥ #	34.335 (0.706) † ‡	32.878 (0.446) ¥ #
Vertical Rectangle HD	13.082 (0.083) † ‡	13.031 (0.076) ¥ #	24.690 (0.268) † ‡	24.316 (0.103) ¥ #	37.266 (1.503) † ‡	34.109 (0.219) ¥ #
Hemisphere HD	11.430 (0.288) ¥ #	12.454 (0.096) ¥ #	17.693 (1.048) ¥ #	23.058 (0.129) ¥ #	20.473 (1.856) ¥ #	31.807 (0.244) ¥ #
Checkered HD	13.541 (0.262) † ‡	14.102 (0.168) #	24.009 (0.322) † ‡	26.065 (0.187) ‡ ¥	31.746 (0.423) † ‡	37.444 (0.295) ‡ ¥
Half-hemisphere HD	12.195 (0.405) ¥	13.332 (0.076) ¥ #	20.625 (0.163) ¥ #	24.678 (0.118) ¥ #	28.039 (0.245) ‡ ¥	34.887 (0.252) ¥ #
Horizontal Rectangle HD	11.188 (0.266) ¥ #	11.332 (0.390) ¥ #	20.048 (0.185) ¥ #	20.960 (1.109) ¥ #	26.141 (0.503) ¥ #	29.670 (0.546) ¥ #
Horizontal Line HD	10.387 (0.913) ¥ #	12.061 (0.135) ¥ #	19.291 (0.269) ¥ #	21.181 (0.762) ¥ #	24.987 (0.463) ¥ #	29.387 (0.795) ¥ #

† Significantly greater torque than smooth socket, p < 0.0005.

‡ Significantly greater torque than OSS socket, p < 0.0005.

¥ Significantly smaller torque than smooth socket, p < 0.0005.

# Significantly smaller torque than OSS socket, p < 0.0005.
