# Supplementary material for: Using mechanical testing to assess texturing of prosthetic sockets to improve suspension in the transverse plane and reduce rotation
Source: PLoS One. 2020 Jun 11;15(6):e0233148. doi: 10.1371/journal.pone.0233148 (PMC7289418; doi:10.1371/journal.pone.0233148)
Supplement: S1 Appendix — (PDF) [file pone.0233148.s001.pdf]

S1 Appendix: Statistical Analysis Results

Table 1. **Three-way mixed ANOVA (BWW) two-way interaction results. Two-way interaction between suspension condition and rotation angle for torque in the transverse plane** (LS: light and sparse; HD: heavy and dense).

**Socket Sample**	**Two-way interaction between suspension and rotation angle**
Smooth Thermoformed	F(1.356, 59.680) = 6963.548, p < .0005, partial η2 = 0.994, ε = .678
Original Squirt-Shape	F(1.402, 61.678) = 8103.924, p < .0005, partial η2 = 0.995, ε = .701
Vertical Line LS	F(1.625, 71.511) = 88.130, p < .0005, partial η2 = 0.667, ε = 813
Vertical Rectangle LS	F(1.082, 47.670) = 159.163, p < .0005, partial η2 = 0.783, ε = .541
Hemisphere LS	F(1.206, 53.048) = 12.711, p < .0005, partial η2 = 0.224, ε = .603
Checkered LS	F(1.343, 59.083) = 211.916, p < .0005, partial η2 = 0.828, ε = .671
Half-Hemisphere LS	F(1.137, 50.003) = 10.619, p = .001, partial η2 = 0.194, ε = .569
Horizontal Rectangle LS	F(1.233, 54.260) = 1894.240, p < .0005, partial η2 = 0.977, ε = .617
Horizontal Line LS	F(1.288, 56.665) = 2181.605, p < .0005, partial η2 = 0.980, ε = .644
Vertical Line HD	F(1.184, 52.083) = 2293.776, p < .0005, partial η2 = 0.981, ε = .592
Vertical Rectangle HD	F(1.012, 44.529) = 254.371, p < .0005, partial η2 = 0.853, ε = .506
Hemisphere HD	F(1.436, 63.182) = 1411.595, p < .0005, partial η2 = 0.970, ε = .718
Checkered HD	F(1.490, 65.558) = 4114.328, p < .0005, partial η2 = .989, ε = .745
Half-Hemisphere HD	F(1.507, 66.320) = 4618.862, p < .0005, partial η2 = 0.991, ε = .754
Horizontal Rectangle HD	F(1.271, 55.910) = 280.592, p < .0005, partial η2 = 0.864, ε = .635
Horizontal Line HD	F(1.692, 74.433) = 124.968, p < .0005, partial η2 = .740, ε = .846

Statistical Analysis Results - continued

Table 2. **Three-way mixed ANOVA (BWW) main effect results.** Main effect of suspension condition on torques for each rotation angle (LS: light and sparse; HD: heavy and dense).

**Socket Sample**	**Simple-simple main effect of suspension on torque for 2.5° rotation**	**Simple-simple main effect of suspension on torque for 5° rotation**	**Simple-simple main effect of suspension on torque for 7.5° rotation**
Smooth Thermoformed	$F(1, 44) = 4777.935, p < .0005$	$F(1, 44) = 11880.172, p < .0005$	$F(1, 44) = 10867.359, p < .0005$
Original Squirt-Shape	$F(1, 44) = 7982.159, p < .0005$	$F(1, 44) = 25498.175, p < .0005$	$F(1, 44) = 20474.984, p < .0005$
Vertical Line LS	$F(1, 44) = 506.171, p < .0005$	$F(1, 44) = 34.201, p < .0005$	$F(1, 44) = 25.820, p < .0005$
Vertical Rectangle LS	$F(1, 44) = 30.283, p < .0005$	$F(1, 44) = 3.923, p = .054$	$F(1, 44) = 279.252, p < .0005$
Hemisphere LS	$F(1, 44) = 569.966, p < .0005$	$F(1, 44) = 140.337, p < .0005$	$F(1, 44) = 3.887, p = .055$
Checkered LS	$F(1, 44) = 349.382, p < .0005$	$F(1, 44) = 1582.978, p < .0005$	$F(1, 44) = 0.00, p = .988$
Half-Hemisphere LS	$F(1, 44) = 39.596, p < .0005$	$F(1, 44) = 194.058, p < .0005$	$F(1, 44) = 14.561, p < .0005$
Horizontal Rectangle LS	$F(1, 44) = 4433.276, p < .0005$	$F(1, 44) = 2765.937, p < .0005$	$F(1, 44) = 2732.261, p < .0005$
Horizontal Line LS	$F(1, 44) = 80.365, p < .0005$	$F(1, 44) = 22391.016, p < .0005$	$F(1, 44) = 16534.035, p < .0005$
Vertical Line HD	$F(1, 44) = 658.060, p < .0005$	$F(1, 44) = 8027.950, p < .0005$	$F(1, 44) = 1295.922, p < .0005$
Vertical Rectangle HD	$F(1, 44) = 1406.710, p < .0005$	$F(1, 44) = 228.760, p < .0005$	$F(1, 44) = 265.652, p < .0005$
Hemisphere HD	$F(1, 44) = 924.318, p < .0005$	$F(1, 44) = 1390.775, p < .0005$	$F(1, 44) = 2012.546, p < .0005$
Checkered HD	$F(1, 44) = 130.740, p < .0005$	$F(1, 44) = 4941.998, p < .0005$	$F(1, 44) = 30923.085, p < .0005$
Half-Hemisphere HD	$F(1, 44) = 366.613, p < .0005$	$F(1, 44) = 23046.629, p < .0005$	$F(1, 44) = 32995.540, p < .0005$
Horizontal Rectangle HD	$F(1, 44) = 5.029, p < .030$	$F(1, 44) = 27.316, p < .0005$	$F(1, 44) = 5053.264, p < .0005$
Horizontal Line HD	$F(1, 44) = 173.485, p < .0005$	$F(1, 44) = 234.562, p < .0005$	$F(1, 44) = 1402.858, p < .0005$

Statistical Analysis Results - continued

Table 3. **Three-way mixed ANOVA (BWW) pairwise comparison results for torque at rotation angle of 2.5° for both suspension conditions** (OV: passive suction with one-way valve; VAC: active vacuum suspension; LS: light and sparse; HD: heavy and dense).

Socket Sample	OV	VAC	Mean Difference (OV-VAC)	Std. Error	Sig.	95% Confidence Interval for Difference	
						Lower Bound	Upper Bound
Smooth Thermoformed	1	2	1.405	.020	.000	1.364	1.446
Original Squirt-Shape	1	2	2.101	.024	.000	2.053	2.148
Vertical Line LS	1	2	.556	.025	.000	.506	.606
Vertical Rectangle LS	1	2	-.653	.119	.000	-.893	-.414
Hemisphere LS	1	2	-.658	.028	.000	-.714	-.603
Checkered LS	1	2	-2.673	.143	.000	-2.961	-2.385
Half-Hemisphere LS	1	2	.585	.093	.000	.398	.772
Horizontal Rectangle LS	1	2	1.332	.020	.000	1.291	1.372
Horizontal Line LS	1	2	1.114	.124	.000	.863	1.364
Vertical Line HD	1	2	.316	.012	.000	.291	.341
Vertical Rectangle HD	1	2	-.051	.001	.000	-.053	-.048
Hemisphere HD	1	2	1.024	.034	.000	.957	1.092
Checkered HD	1	2	.561	.049	.000	.462	.660
Half-Hemisphere HD	1	2	1.137	.059	.000	1.018	1.257
Horizontal Rectangle HD	1	2	.145	.065	.030	.015	.275
Horizontal Line HD	1	2	1.674	.127	.000	1.418	1.930

Statistical Analysis Results - continued

Table 4. **Three-way mixed ANOVA (BWW) pairwise comparison results for torque at rotation angle of 5° for both suspension conditions** (OV: passive suction with one-way valve; VAC: active vacuum suspension; LS: light and sparse; HD: heavy and dense).

Socket Sample	OV	VAC	Mean Difference (OV-VAC)	Std. Error	Sig.	95% Confidence Interval for Difference	
						Lower Bound	Upper Bound
Smooth Thermoformed	1	2	4.820	.044	.000	4.731	4.909
Original Squirt-Shape	1	2	4.627	.029	.000	4.569	4.686
Vertical Line LS	1	2	-1.404	.240	.000	-1.888	-.920
Vertical Rectangle LS	1	2	-.520	.263	.054	-1.050	.009
Hemisphere LS	1	2	-1.053	.089	.000	-1.232	-.874
Checkered LS	1	2	-1.078	.027	.000	-1.133	-1.024
Half-Hemisphere LS	1	2	1.726	.124	.000	1.477	1.976
Horizontal Rectangle LS	1	2	5.805	.110	.000	5.582	6.027
Horizontal Line LS	1	2	4.898	.033	.000	4.832	4.964
Vertical Line HD	1	2	.649	.007	.000	.634	.663
Vertical Rectangle HD	1	2	-.375	.025	.000	-.424	-.325
Hemisphere HD	1	2	5.366	.144	.000	5.076	5.656
Checkered HD	1	2	2.056	.029	.000	1.997	2.115
Half-Hemisphere HD	1	2	4.053	.027	.000	4.000	4.107
Horizontal Rectangle HD	1	2	.912	.175	.000	.561	1.264
Horizontal Line HD	1	2	1.890	.123	.000	1.641	2.138

Statistical Analysis Results - continued

Table 5. **Three-way mixed ANOVA (BWW) pairwise comparison results for torque at rotation angle of 7.5° for both suspension conditions** (OV: passive suction with one-way valve; VAC: active vacuum suspension; LS: light and sparse; HD: heavy and dense).

Socket Sample	OV	VAC	Mean Difference (OV-VAC)	Std. Error	Sig.	95% Confidence Interval for Difference	
						Lower Bound	Upper Bound
Smooth Thermoformed	1	2	9.425	.090	.000	9.243	9.607
Original Squirt-Shape	1	2	8.529	.060	.000	8.409	8.649
Vertical Line LS	1	2	1.164	.229	.000	.702	1.626
Vertical Rectangle LS	1	2	4.853	.290	.000	4.267	5.438
Hemisphere LS	1	2	-.288	.146	.055	-.582	.006
Checkered LS	1	2	0.001	.077	.988	-.154	.157
Half-Hemisphere LS	1	2	1.497	.392	.000	.707	2.288
Horizontal Rectangle LS	1	2	13.968	.267	.000	13.430	14.507
Horizontal Line LS	1	2	9.936	.077	.000	9.780	10.091
Vertical Line HD	1	2	-1.458	.040	.000	-1.539	-1.376
Vertical Rectangle HD	1	2	-3.157	.194	.000	-3.547	-2.767
Hemisphere HD	1	2	11.334	.253	.000	10.825	11.843
Checkered HD	1	2	5.698	.032	.000	5.633	5.763
Half-Hemisphere HD	1	2	6.848	.038	.000	6.772	6.924
Horizontal Rectangle HD	1	2	3.528	.050	.000	3.428	3.628
Horizontal Line HD	1	2	4.400	.117	.000	4.164	4.637
